# Different Within-Host Viral Evolution Dynamics in Severely Immunosuppressed Cases with Persistent SARS-CoV-2

**DOI:** 10.3390/biomedicines9070808

**Published:** 2021-07-13

**Authors:** Laura Pérez-Lago, Teresa Aldámiz-Echevarría, Rita García-Martínez, Leire Pérez-Latorre, Marta Herranz, Pedro J. Sola-Campoy, Julia Suárez-González, Carolina Martínez-Laperche, Iñaki Comas, Fernando González-Candelas, Pilar Catalán, Patricia Muñoz, Darío García de Viedma

**Affiliations:** 1 Clinical Microbiology and Infectious Diseases Department, Gregorio Marañón General University Hospital, 28007 Madrid, Spain; lperezg00@gmail.com (L.P.-L.); teresaldamiz@yahoo.es (T.A.-E.); legor78@hotmail.com (L.P.-L.); m_herranz01@hotmail.com (M.H.); pedroscampoy@gmail.com (P.J.S.-C.); pilar.catalan@salud.madrid.org (P.C.); pmunoz@hggm.es (P.M.); 2 Gregorio Marañón Health Research Institute (IiSGM), 28007 Madrid, Spain; humrita77@yahoo.es (R.G.-M.); julia.suarez.glez@gmail.com (J.S.-G.); cmlaperchehgugm@gmail.com (C.M.-L.); 3 Internal Medicine Department, Gregorio Marañón General University Hospital, 28007 Madrid, Spain; 4 CIBER Respiratory Diseases (CIBERES), 28029 Madrid, Spain; 5 Genomics Unit, Gregorio Marañón General University Hospital, 28007 Madrid, Spain; 6 Oncohematology Service, Gregorio Marañón General University Hospital, 28007 Madrid, Spain; 7 Tuberculosis Genomics Unit, Instituto de Biomedicina de Valencia-CSIC, 46010 Valencia, Spain; icomas@ibv.csic.es; 8 CIBER Salud Pública (CIBERESP), 28029 Madrid, Spain; fernando.gonzalez@uv.es; 9 Joint Research Unit “Infection and Public Health”, Institute for Integrative Systems Biology (I2SysBio), FISABIO-University of Valencia, 46980 Valencia, Spain; 10 Departamento de Medicina, Universidad Complutense, 28040 Madrid, Spain

**Keywords:** COVID-19, SARS-CoV-2, persistence, immunosuppressed, diversity, viral viability, genomics

## Abstract

A successful Severe Acute Respiratory Syndrome Coronavirus 2 (SARS-CoV-2) variant, B.1.1.7, has recently been reported in the UK, causing global alarm. Most likely, the new variant emerged in a persistently infected patient, justifying a special focus on these cases. Our aim in this study was to explore certain clinical profiles involving severe immunosuppression that may help explain the prolonged persistence of viable viruses. We present three severely immunosuppressed cases (A, B, and C) with a history of lymphoma and prolonged SARS-CoV-2 shedding (2, 4, and 6 months), two of whom finally died. Whole-genome sequencing of 9 and 10 specimens from Cases A and B revealed extensive within-patient acquisition of diversity, 12 and 28 new single nucleotide polymorphisms, respectively, which suggests ongoing SARS-CoV-2 replication. This diversity was not observed for Case C after analysing 5 sequential nasopharyngeal specimens and one plasma specimen, and was only observed in one bronchoaspirate specimen, although viral viability was still considered based on constant low Ct values throughout the disease and recovery of the virus in cell cultures. The acquired viral diversity in Cases A and B followed different dynamics. For Case A, new single nucleotide polymorphisms were quickly fixed (13–15 days) after emerging as minority variants, while for Case B, higher diversity was observed at a slower emergence: fixation pace (1–2 months). Slower SARS-CoV-2 evolutionary pace was observed for Case A following the administration of hyperimmune plasma. This work adds knowledge on SARS-CoV-2 prolonged shedding in severely immunocompromised patients and demonstrates viral viability, noteworthy acquired intra-patient diversity, and different SARS-CoV-2 evolutionary dynamics in persistent cases.

## 1. Introduction

A recent review [1] established that the mean duration of SARS-CoV-2 in upper respiratory tract viral shedding is 17 days, with a maximum of 83 days, although certain studies report SARS-CoV-2 positive RT-PCRs up to 101 days [2]. With the identification of prolonged persistence, it was necessary to clarify whether these cases were still infectious or if they corresponded to the presence of residual genomic RNA. The standard approach to assess viability has relied on cell cultures, and most studies fail to detect live viruses beyond Day 9 [1]. The detection of subgenomic viral RNA as a way to infer viral replication has been used as an alternative to cell cultures to assess viability [2].

The dynamics of SARS-CoV-2 are a balance between its replication and the host’s immune response to control it. Thus, immunocompromised patients may offer the virus better opportunities to persist. This subgroup of patients may constitute a suitable model to dissect the ability of SARS-CoV-2 to acquire intrapatient diversity, determine the magnitude of intrahost evolution, evaluate the potential role of acquired mutations, and extend our understanding on the dynamics of SARS-CoV-2 infection.

Whole genome sequencing of SARS-CoV-2 is key to understand the global expansion of the virus [3], track current transmission dynamics [4] or outbreaks [5], and determine reinfections [6]. However, the genomic analysis has scarcely been applied to better understand the evolutionary dynamics of SARS-CoV-2 along persistent infections [7,8,9]. The interest of understanding within-patient evolution of SARS-CoV-2 in prolonged infections has recently increased due to the identification of a successful variant in the UK, B.1.1.7, which has been hypothesized to have emerged from one persistently infected case [10].

In this study, we present a detailed clinical description of three severely immunocompromised subjects with SARS-CoV-2 prolonged shedding, in-depth genomic viral analysis on multiple sequential specimens, and an assessment of viability in cell cultures.

## 2. Materials and Methods

### 2.1. Diagnostic RT-PCR

RNA was extracted and purified from 300 µL of a nasopharyngeal exudate with the KingFisher (Thermo Fisher Scientific, Waltham, MA, USA), and the TaqPath COVID-19 CE-IVD RT-PCR (Thermo Fisher Scientific, Waltham, MA, USA) was applied.

### 2.2. Whole Genome Sequencing

Viral sequencing was done using the Artic_nCov-2019_V3 panel of primers (Integrated DNA Technologies, Inc., Coralville, IA, USA) (artic.network/ncov-2019, 24 March 2020), and libraries were prepared using the Nextera Flex DNA Library Preparation Kit (Illumina lnc, San Diego, CA, USA) and sequenced on the Miseq system (Illumina Inc, San Diego, CA, USA). Details for WGS and bioinformatic analysis are specified in Supplementary Materials. Fasta files above the GISAID thresholds were deposited in GISAID (accession numbers in Supplementary Materials).

The following frequencies were considered for variant calling: minority variants (MVs; frequency < 20%), intermediate variants (IVs; frequency 20–80%), and fixed single nucleotide polymorphisms (SNPs; frequency > 80%).

### 2.3. Short Tandem Repeat Analysis

Human identity testing analysis was performed by short tandem repeat (STR) PCR (Mentype^®^ Chimera^®^ Biotype, Dresden, Germany) on the same specimens used to perform SARS-CoV-2 RT-PCRs and sequencing. Details are included in Supplementary Materials.

### 2.4. Cell Cultures

For viral amplification, Vero E6 cells (ATCC CRL1586) were inoculated with positive SARS-CoV-2 samples. Details are included in Supplementary Materials.

Cultures were considered positive when fulfilling at least one of the following criteria: (i) appearance of CPE, (ii) positive immunofluorescence detected, or (iii) a decrease of ≥3 (equivalent to a 1 log increase in virus quantity) between the Ct of the original sample and final culture supernatant. 

### 2.5. Subgenomic RNA

RNA remnants purified (KingFisher, Thermo Fisher Scientific, Waltham, MA, USA) from diagnostic nasopharyngeal swab specimens (300 µL of UTM swabs, COPAN, Biomerieux, Marcy-l’Étoile, France) were used as templates for the PCR design described elsewhere [11] for specific detection of the SG *E* gene RNA (+strand).

## 3. Results

### 3.1. Case A

#### 3.1.1. Clinical Case

**Figure 1:**
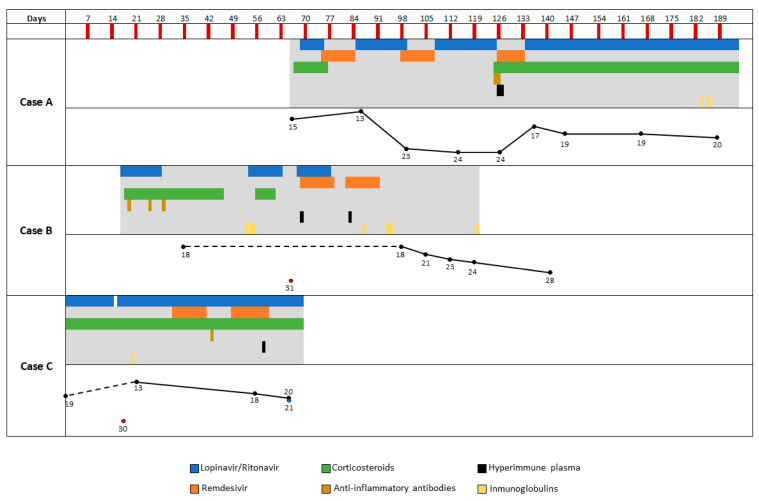
Treatment history for the study cases. Day 0 corresponds to diagnosis. For Case A, only the therapies administered during the period he was managed in our institution are indicated. Anti-inflammatory antibodies refer to either tozilizumab or anakinra. The graphs at the lower part of each case´s panel represent the Ct values for the positive RT-PCRs along the infection.

A 52-year-old male with rituximab treatment every three months for a follicular lymphoma in remission was admitted in June 2020. SARS-CoV-2 infection was diagnosed on 23 March (Day 0) 2020, a few days after receiving the last anti-CD20 treatment. For the first months of infection, the patient had been admitted to another hospital, where he presented intermittent fever and several episodes of pulmonary pneumonia, receiving treatment with dexamethasone, hyperimmune plasma, anakinra, hydroxychloroquine, and azithromycin. Once discharged and under a dexamethasone tapering schedule, the fever reappeared, and he came to our unit (Day 66 from diagnosis). Although febrile, he was in good condition with no respiratory symptoms nor radiological alterations. The SARS-CoV-2 nasopharyngeal PCR test was positive, and the serological test for coronavirus was negative. Corticosteroids were ruled out, and the patient was started on lopinavir/ritonavir (Figure 1). A few days later the chest X-Ray showed bilateral linear opacities. Lopinavir/ritonavir was switched to remdesivir for ten days. Once that treatment ended, the patient was restarted on lopinavir/ritonavir, adding hydroxychloroquine. From Days 71 to 126 the subject experienced intermittent fever and worse radiological findings. He completed two additional remdesivir courses. On Day 126 his clinical condition worsened, developing respiratory failure, and he was admitted to the intensive care unit (ICU) for noninvasive respiratory therapy. He was restarted on corticosteroids, received hyperimmune plasma, tocilizumab, and retreatment with remdesivir (Figure 1). On Day 144 he was discharged from the ICU and readmitted in the Infectious Diseases ward, maintaining corticosteroid and lopinavir/ritonavir therapies and supplementary oxygen. A few days later he was discharged, and that same therapy was prescribed at home. On Day + 167, he began to experience increasing dyspnoea and worsening radiological infiltrates despite increasing doses of steroids, which lead to a severe respiratory failure with no response to invasive respiratory supplies. The serological test for SARS-CoV-2 was still negative, and nasopharyngeal PCR was positive at low levels. Sputum microbiological analyses showed high positivity for *Aspergillus flavus* in cultures. The patient died on Day 194.

#### 3.1.2. Microbiological Analysis

**Figure 2:**
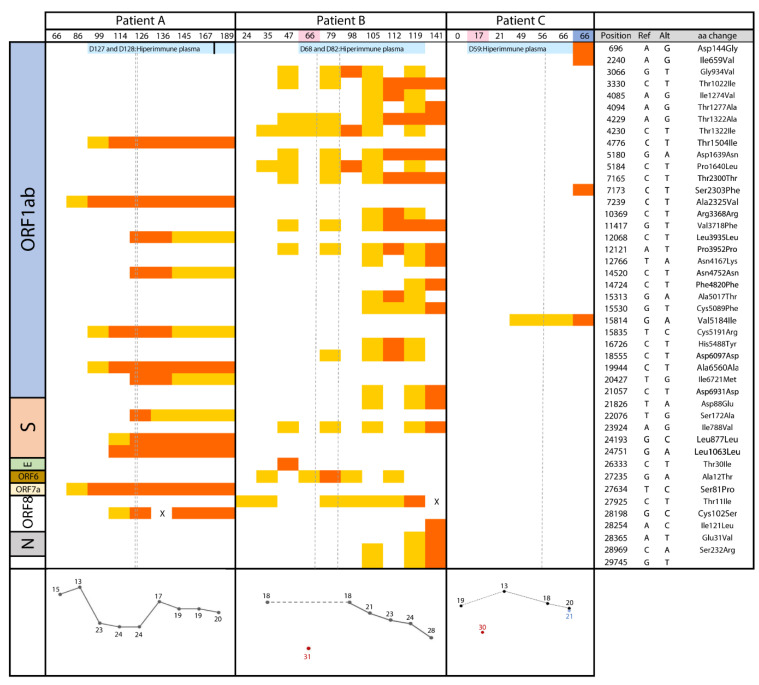
Chronological acquisition of diversity for the three study cases (days are indicated at the upper file; days coloured in pink correspond to plasma, in blue to bronchoaspirate, and without colour to nasopharyngeal specimens). On the left side, the SARS-CoV-2 genetic content is indicated. Single nucleotide polymorphisms are indicated in orange, when identified as fixed, and in yellow when they are found as minority/intermediate variants. In the right panel, the coordinates and specific substitutions are shown (nonsynonymous changes are highlighted in bold). At the bottom panel, Ct values for the specimens used in the sequencing analysis are shown. Dots out of the lines correspond to specimens other than nasopharyngeal (plasma and bronchoaspirate). All sequencing data covered >96% of the reference genome, with a 100X depth. The positions with an “X” correspond to the only 2 positions involved in the diversity analysis, which were not sufficiently covered in two specimens among those in the study.

SARS-CoV-2 sequences were obtained from nine positive RT-PCR nasopharyngeal specimens (between 28 May and 28 September 2020; Days 66, 86, 99, 114, 126, 136, 145, 167, and 189; Ct values 13–24; Figure 2). Short-tandem-repeats analysis confirmed that all specimens were collected from the same patient. SARS-CoV-2 subgenomic RNA, suggesting viral replication, was detected in all nine sequential specimens (Supplementary Table S1).

No samples were available for the first stages of his persistent infection (23 March–28 May) because the patient was managed at another institution. 

The strain on the first day (Day 66) corresponded to the B/19A lineage and showed six SNPs with respect to the Wuhan-1 reference strain (Supplementary Table S1). From Day 66 to the last collected specimen on Day 189, fifteen SNPs were found as ephemeral minority variants/intermediate variants (MV/IVs; 2–3 at each specimen with estimated frequencies in the 11–78% range); all, except one, were detected in one of the specimens and then cleared (Supplementary Table S2). Between Day 66 and Day 126, twelve SNPs not present in the first analysed specimens emerged and were fixed, while another SNP initially present (94%) was cleared. This adds a total number of 18 SNPs with respect to the Wuhan-1 reference sequence.

Among the acquired SNPs, seven were nonsynonymous substitutions and mapped in genes *S* (one), open reading frame (ORF) *1ab* (four), *ORF 7a* (one), and *ORF 8a* (one) (Figure 2). Seven of the twelve intrapatient-fixed SNPs were detected as MVs or IVs (11-67%) in a previous sample and were rapidly fixed in the next specimens (12–15 days after being detected for the first time). Regarding the remaining five SNPs, the fixation seemed to occur even faster, as they appeared directly on Day 114 and 126 and were absent (0%) in the specimens taken just a few days before (Figure 2).

On Day 126 the patient got worse and received hyperimmune plasma for the first time (Days 127 and 128). From then on, a different evolutionary pattern was observed (Supplementary Table S2). Nine additional MVs and IVs emerged, but did not overpass the minimum frequency threshold to be considered fixed, and, finally, no new additional SNP was fixed. Moreover, the four SNPs which had been detected as almost fixed on day 126 reduced their frequency in the four subsequent specimens, collected between Day 126 and Day 189 (from 93–95% to 47–76% of the total calls).

### 3.2. Case B

#### 3.2.1. Clinical Case

A 47-year-old male with follicular lymphoma on treatment with rituximab-bendamustine (last cycle on 4 March 2020) developed persistent lymphopenia (lymphocytes < 0.5 10^3^/µL).

The patient started with a cough and fever on 21 March 2020 and had a positive SARS-CoV-2 RT-PCR on 23 March (Day 0). Since he had no pneumonia, he quarantined at home and received hydroxychloroquine for 10 days.

Eighteen days after the onset of symptoms, he was admitted to a hospital because of pneumonia, starting on lopinavir/ritonavir (12 days). After ruling out other infections, high doses of dexamethasone (40 mg/day, five days) and tocilizumab (single dose 600 mg) were administered because of clinical worsening (Figure 1). On Day 24 he needed noninvasive respiratory support and treatment intensification with high doses of dexamethasone and anakinra (100 mg/six hours, two days), followed by tapering of steroids. He had a transient improvement and was descaled from noninvasive respiratory support on Day 37.

On Day 47 he developed fever and progressive respiratory failure; SARS-CoV-2 RT-PCR was positive. On day 54, steroids were increased, lopinavir/ritonavir plus hydroxychloroquine (10 days) were restarted, and nonspecific immunoglobulin was prescribed together with antibiotics and antifungal prophylaxis (Figure 1). He required non-invasive respiratory support again and was admitted to the ICU. He persisted with severe respiratory failure, lymphopenia (lymphocytes < 0.1 10^3^/µL), and positive RT-PCR. Remdesivir+lopinavir/ritonavir (for 10 days) and hyperimmune plasma (single dose) were started (Days 68 and 69). Given the clinical improvement, he was discharged from the ICU on Day 77. Two days after ending antivirals, he developed fever, and RT-PCR was persistently positive. Remdesivir plus hyperimmune plasma were restarted on Day 82, followed by nonspecific immunoglobulin (30 g). A transient positive serology (days 74 to 80) for SARS-Cov-2 was observed, which became negative at Day 94 and remained thereafter. He showed steady respiratory improvement and was discharged from hospital on Day 120. His nasopharyngeal swab was negative on Day 195 from symptoms onset.

Immunological and biochemical data along the infection are compiled in the Table 1.

#### 3.2.2. Microbiological Analysis

SARS-CoV-2 sequences were obtained from nine nasopharyngeal specimens and one plasma positive RT-PCR specimen (16 April–11 August 2020; (Days 24, 35, 47, 66 (plasma), 79, 98, 105, 112, and 141); Ct values 18-28 were obtained for nasopharyngeal specimens and 31 for plasma (Figure 2). STR analysis confirmed that all the specimens were collected from the same patient. Subgenomic RNA was detected in all sequential nasopharyngeal specimens (Supplementary Table S1). Viral viability on cell culture was observed for five NP specimens, corresponding to Days 24, 35, 47, 79, and 112 (Supplementary Table S1).

The strain corresponded to lineage B.1.5/20A and showed five SNPs with respect to the Wuhan-1 strain, including the prevalent D614G substitution, affecting the SARS-CoV-2 spike glycoprotein, which surfaced in southern Europe (Supplementary Table S3).

From Day 24 to the last collected specimen on Day 141, 62 transient MVs or IVs (4–16 at each specimen with a frequency ranging between 11–79%) were identified; most were detected in one of each of the analysed specimens, and only three were detected in more than three specimens (Supplementary Table S3). The number of transient variants identified in a single nasopharyngeal specimen was higher from Day 79 on (Supplementary Table S3), coinciding with the worsening of the patient. Thus, steroids (to high doses), antivirals, and antibiotics were increased. The specimen of Day 98 showed four fixed SNPs with reduced frequency in the following specimens that finally cleared (Figure 2). From day 112 and on, we observed new fixed SNPs. Eleven SNPs were found on Day 112, five of them later cleared, and another eleven on Day 141, adding a total of 17 new SNPs in the last specimen. All but one among the newly acquired SNPs were preceded by stages in which they were first detected as MV/IVs. Only for a few SNPs was the process of fixation after appearing as an MV as fast as in Case A; most persisted as MVs/IVs in several sequential specimens (2–6; 1–3 months) before acquiring a frequency above 80% in the viral population. Among the 17 intrapatient SNPs, 12 were nonsynonymous changes and mapped in ORF1ab (seven), ORF 8 (one), S (two), and N (two) (Figure 2).

The plasma specimen was the one with the highest number of variants (17, mostly transient); only two were also found in some nasopharyngeal specimens. One fixed SNP was exclusively detected in blood and not in nasopharyngeal specimens.

### 3.3. Case C

#### 3.3.1. Clinical Case

A 63-year-old female with Ellis-Van-Creveld syndrome and grade IV follicular lymphoma in complete remission after undergoing treatment until a year ago with rituximab and bendamustine. Secondary to this treatment, the patient presented cellular and humoral immunodeficiency and persistent lymphopenia (lymphocytes: 0.2 10^3^/µL) requiring periodic administration of intravenous immunoglobulins. Last year, she was admitted on several occasions due to fever and pancytopenia concordant with a hemophagocytic syndrome without a clear trigger and for which she received oral corticosteroids.

The patient was admitted on Day 23 due to fever and pancytopenia. SARS-CoV-2 RT-PCR was negative; prednisone was increased from 5 to 15 mg per day with improvement. On Day -2 she again presented fever; on Day 0 SARS-CoV-2 RT-PCR was positive and pneumonia was observed, so lopinavir/ritonavir (D0–D14), hydroxychloroquine (D0-D11), and ceftriaxone were started with clinical improvement (Figure 1). On Day 15, she again presented dyspnoea and worsening of pulmonary infiltrates; lopinavir/ritonavir, azithromycin, and ertapenem were restarted on Day 16 with good evolution. On Day 20, intravenous immunoglobulins were administered as a part of her regular treatment. On Day 30, she again developed dyspnoea and bilateral pulmonary infiltrates. Remdesivir (D32–D41), corticosteroids at higher doses (40 mg of methylprednisolone daily), and high-flux oxygen therapy (D35-D45) were added to the lopinavir/ritonavir treatment (Figure 1). On Day 38 the patient presented mild mixed aphasia that progressed to severe in the subsequent days. A new cycle of remdesivir was administered in addition to lopinavir/ritonavir. On Day 58, hyperimmune plasma was administered without clinical response. On Day 64 the patient presented an intestinal obstruction that required surgery, after which the patient died on Day 69 in the ICU.

Immunological and biochemical data along the infection are compiled in the Table 1.

#### 3.3.2. Microbiological Analysis

SARS-CoV-2 sequences were obtained from seven specimens (27 April–2 July 2020: five nasopharyngeal (Days 0, 21, 49, 56, and 66), one bronchoaspirate (Day 66), and one plasma (Day 17): Ct values 13-2, Figure 2). Viral viability on cell cultures was observed for the two last specimens taken on Day 66 (Supplementary Table S1). Subgenomic RNA, suggesting actively replicating viruses, was detected in the specimens taken on Days 49, 56, and 66 (Supplementary Table S1). STR analysis confirmed that all specimens were collected from the same patient. 

The strain corresponded to lineage A.5/19B, and ten SNPs were found with respect to the Wuhan-1 strain (Supplementary Table S4). Eight transient MV/IVs were identified (0–5 in each specimen with a 12–44% frequency) from the collected nasopharyngeal specimens throughout the persistence, one in each specimen, except for two MVs detected in two specimens. The first specimen harboured no variants, and the last two specimens (nasopharyngeal and bronchoaspirate), three days before Case C died, accumulated 30 and 4 MV/IVs, respectively (Supplementary Table S4). Only eight variants were present, at intermediate frequencies, in the last two-three nasopharyngeal specimens, although neither finally fixed. However, in the bronchoaspirate specimen taken the same day as the last nasopharyngeal swab, one of these IVs appeared as fixed and, additionally, three more SNPs appeared as fixed and another two appeared at high frequencies; none of them had been detected in the nasopharyngeal specimens. The four fixed SNPs were nonsynonymous changes all mapping in the ORF1ab (Figure). The plasma specimen showed six MV/IVs not detected in respiratory specimens.

## 4. Discussion

SARS-CoV-2 replication is the result of a complex balance between the virus, the host’s immune response, and the prescribed antiviral therapy. Thus, severely immunosuppressed patients may constitute exceptions to the current average time references for SARS-CoV-2 persistence [1,2].

Here, we analysed three cases of long-term viable SARS-CoV-2 shedders (beyond the times reported for similar cases elsewhere). To the best of our knowledge, for one of the cases, the persistence of viable SARS-CoV-2 was the longest reported to date: 189 days. These cases offered us an opportunity to dissect their within-host genome evolutionary dynamics and viral viability. In all three cases, we found evidence for sustained viral replication throughout the course of the disease, and therefore potential contagiousness. We support this assumption on permanent low Cts, acquisition of SNPs leading to a continuously evolving within-host diversity, detection of subgenomic viral RNA, and the assessment of viral viability in cell cultures.

The three patients in this study presented severe cellular and humoral immunosuppression secondary to a previous diagnosis of follicular lymphoma and received rituximab and bendamustine treatment. Rituximab causes depletion of B lymphocytes, and its effect may last six months or more. This deficiency of B lymphocytes may determine an innate humoral and memory-deficient response to SARS-CoV-2. In fact, none of the three patients had antibodies against SARS-CoV2 at the end of follow-up. These were only detected transiently in case B and after the administration of hyperimmune plasma, suggesting the detection of exogenous antibodies and not a specific immune response. On the other hand, bendamustine causes depletion of T lymphocytes, and its effect may last up to nine months. T lymphocytes play a key role in the presentation of the pathogen´s antigen necessary to trigger the host’s immune response and in the destruction of infected cells through memory response. Considering this, it is possible that the impaired immunity of the patients included in this work explained the persistence of the infection. Moreover, the three patients showed severe absolute lymphopenia that lasted for months and was already present before being infected by the SARS CoV-2, and immunological and biochemical markers (Table) indicated they had a profound deregulation of the immune system.

The three patients presented differences regarding the acquisition of within-host viral diversity. For cases A and B, we observed a remarkable acquisition of diversity; within-host acquisition of diversity by SARS-CoV-2 in persistent cases has recently received additional relevance. The successful B.1.1.7 variant reported in southeast England, carrying a usual number of genetic changes [12], has been proposed to have emerged in a persistently infected case.

For Case C, the within-host acquisition of viral diversity was much more moderate. There is no clear explanation for such different viral dynamics behaviour. If we analyse their antiviral treatment, all three patients received remdesivir and lopinavir/ritonavir during worsening clinical episodes. Furthermore, Case A exhibited a unique behaviour, namely, a slowdown in the acquisition of diversity during the last period of persistence, coinciding with the administration of hyperimmune plasma, which in other viruses may have an effect in decreasing viral load [13]. However, this was not observed in Case B, for which constant acquisition of diversity occurred, even after receiving hyperimmune plasma. Shifts in viral dynamics after the patient was given convalescent plasma have been reported, with minor evolutionary changes before the administration [14,15].

Regarding immunosuppression, the major difference between the two cases with marked viral diversity and the one without it is that the two first had recently received anti-CD20 treatment, only days before the onset of COVID-19 symptoms, while Case C had received it one year ago. Although we cannot stablish a strong relationship between the recent administration of anti-CD20 and the more marked acquisition of viral diversity in cases A and B, a more intense immunosuppression, secondary to the anti-CD20 treatment itself, might be expected for these two cases, which could justify a less efficient viral containment and therefore a higher acquisition of diversity. Moreover, the three patients received immunosuppressive treatment during the course of the SARS-CoV2 infection; however, Case B received a larger number of immunosuppressive drugs (pulses of corticosteroids, anakinra, and tocilizumab), and Case C only received doses of corticosteroids that were somewhat higher than usual.

We observed differences in the rate of acquisition of diversity, 12 and 28 SNPs, between the two shedders with marked viral diversity. The rate of SNP acquisition for SARS-CoV-2 is one SNP/10–15 days [16]. Thus, the diversity we observed for Case A fits with these expectations, while Case B exceeded this rate. Case A also showed transient MV/IVs to a higher extent, which were fixed shortly after they appeared, while in Case B a longer time was required for a variant to be fixed. Differences in the dynamics of SARS-CoV-2 evolution in persistent COVID-19 cases are found elsewhere [7,8,9].

It has to be noted that the SARS-CoV-2 strain infecting Case B carried the spike protein substitution D614G, associated with increased infectivity in cellular and animal models [17]. In previous reports, most within-patient acquired SNPs mapped on gene *S*, mainly at the receptor binding domain, suggesting a potential role for these changes [9]. In our cases, SNPs were distributed along different ORFs, which limits the interpretation of a functional relevance from our data.

Compartmentalization of coexisting SARS-CoV-2 variants in a single patient has been described [18,19]. A high number of MV/IVs were found in a plasma specimen collected from Case B, some of them not identified in respiratory specimens, suggesting viral diversity also in blood. Similar compartmentalization was observed for Case C, where the only fixed variants were found in a bronchoaspirate specimen, although a nasopharyngeal specimen had been also collected on the same day.

We report three cases that increase the currently limited number of descriptions of prolonged SARS-CoV-2 shedders, for which viral diversity characterized by WGS [7,8,9] has been performed, and offer valuable new insights on SARS-CoV-2 prolonged shedding in immunocompromised patients, more specifically those receiving anti-CD20 treatment. Sequential genomic analysis and identification of acquisition of diversity may constitute an alternative to cell culture to allow inferring ongoing viral replication and viability in SARS-CoV-2 and, therefore, contagiousness. Our data point to a complexity behind COVID-19 long shedders, with no common pattern for the SARS-CoV-2 evolution. The viral dynamics in relation to the clinical evolution and antiviral management were not obvious. Similar therapeutic interventions led either to a decrease in the rate of diversity acquisition or had no impact on it, suggesting the existence of additional, still unknown roles for either the host or viral associated factors. The study of within-patient viral evolution in persistent immunocompromised COVID-19 long shedders deserves further attention after the recent description that the successful variant B.1.1.7 might have emerged from one of these cases. 

## Figures and Tables

**Table 1:** Clinical values of patients along the infection. (Reference values: Linfocytes (1.2–5.2), D-Dimer (0–250), LDH (135–225), C-Reactive Protein (0.0–0.5), Ferritin (22–274), IL-6 < 4.3, CD4 (300–1400), IgG (650–1600)).

**Case A**	**27/May–15/June**	**15/June–30/June**	**01/July–15/July**	**16/July–30/July**	
Lymphocytes (×10^3^/µL)	300	700	1100	600	
D-dimer (ng/mL)	309	286	233	378	
LDH (U/L)	262	280	303	418	
C- reactive protein (mg/dL)	3.2	8.3	15.9	22.4	
Ferritin (µg/L)	4797	3683	7504	7433	
IL-6 (pg/mL)	20	20.5	56.3	335	
CD4 (cells/µL)	86 (23%)			175	
IgG (mg/dL)	545	517	429		
SARS-CoV-2 Serology	Negative	Negative	Negative	Negative	
**Case B**	**8/April**	**14/May**	**12/August**	**20/October**	
Lymphocytes (×10^3^/µL)	100	100	400	500	
D-dimer (ng/mL)	581	168			
LDH (U/L)	374	326	403		
C- reactive protein (mg/dL)	4.1	10.3	0.5	0.3	
Ferritin (µg/L)	4559	2723	575	50	
IL-6 (pg/mL)	44.1	88.5			
CD4 (cells/µL)	17 ( 16%)	9 (27.3%)	106(25.7%)	109(20.8%)	
IgG (mg/dL)	578	353	1070	760	
SARS-CoV-2 Serology		Negative	Negative	Negative	
**Case C**	**3/April–30/April**	**1/May–14/May**	**15/May–31/May**	**1/June–15/June**	**5/July**
Lymphocytes (×10^3^/µL)	200	300	100	200	100
D-dimer (ng/mL)		185	1007	837	384
LDH (U/L)	203	268	350	246	199
C- reactive protein (mg/dL)	5	3.7	5.3	4.5	14.3
Ferritin (µg/L)	2349	2655	2637	4940	
IL-6 (pg/mL)	38.4	56.1	108.4		
CD4 (cells/µL)	45				
IgG (mg/dL)	917			605	
SARS-CoV-2 Serology				Negative	Negative

## Data Availability

Fasta files were deposited in GISAID.

## Supplementary Materials

The following are available online at https://www.mdpi.com/article/10.3390/biomedicines9070808/s1, Supplementary Methods: Detailed description of the study methodology, Supplementary Table S1: Result of cell cultures and subgenomic RNA assays, Supplementary Table S2: Allelic frequencies of the variants present in the virus genome along the infection of patient A, Supplementary Table S3: Allelic frequencies of the variants present in the virus genome along the infection of patient B, Supplementary Table S4: Allelic frequencies of the variants present in the virus genome along the infection of patient C.
