# Supplementary material for: Different Within-Host Viral Evolution Dynamics in Severely Immunosuppressed Cases with Persistent SARS-CoV-2
Source: Biomedicines. 2021 Jul 13;9(7):808. doi: 10.3390/biomedicines9070808 (PMC8301427; doi:10.3390/biomedicines9070808)
Supplement: Supplementary file 1 [file biomedicines-09-00808-s001.zip › biomedicines-1250640-SI/Sumpplementary Table_S3.pdf]

# Patient B

| | Nasopharyngeal | asopharynge | Nasopharyngeal | Plasma | Nasopharyngeal | Nasopharyngeal | Nasopharyngeal | Nasopharyngeal | Nasopharyngeal | Nasopharyngeal | | | |
|---|---|---|---|---|---|---|---|---|---|---|---|---|---|
| | 26 | 37 | 49 | 68 | 81 | 100 | 107 | 114 | 121 | 143 | | | |
| | 04/16/2020 | 04/27/2020 | 05/09/2020 | 05/28/2020 | 06/10/2020 | 06/10/2020 | 07/06/2020 | 07/13/2020 | 07/20/2020 | 08/11/2020 | Annotation | Gen | a.a change |
| C\|2973\|T | 0.13 | 0.0 | 0.0 | 0.0 | 0.0 | 0.0 | 0.0 | 0.0 | 0.0 | 0.0 | | | |
| G\|5206\|A | 0.13 | 0.0 | 0.0 | 0.0 | 0.0 | 0.0 | 0.0 | 0.0 | 0.0 | 0.0 | | | |
| C\|6781\|T | 0.0 | 0.13 | 0.0 | 0.0 | 0.0 | 0.0 | 0.0 | 0.0 | 0.0 | 0.0 | | | |
| C\|8752\|T | 0.25 | 0.0 | 0.0 | 0.0 | 0.0 | 0.0 | 0.0 | 0.0 | 0.0 | 0.0 | | | |
| C\|5178\|T | 0.0 | 0.0 | 0.0 | 0.23 | 0.0 | 0.0 | 0.0 | 0.0 | 0.0 | 0.0 | | | |
| G\|27358\|T | 0.0 | 0.2 | 0.0 | 0.0 | 0.0 | 0.0 | 0.0 | 0.0 | 0.0 | 0.0 | | | |
| G\|28230\|T | 0.0 | 0.22 | 0.0 | 0.0 | 0.0 | 0.0 | 0.0 | 0.0 | 0.0 | No reads | | | |
| T\|9199\|C | 0.0 | 0.0 | 0.0 | 0.0 | 0.12 | 0.0 | 0.0 | 0.0 | 0.0 | 0.0 | | | |
| C\|20628\|T | 0.0 | 0.0 | 0.11 | 0.0 | 0.0 | 0.0 | 0.0 | 0.0 | 0.0 | 0.0 | | | |
| G\|4232\|A | 0.0 | 0.0 | 0.0 | 0.11 | 0.0 | 0.0 | 0.0 | 0.0 | 0.0 | 0.0 | | | |
| A\|4233\|G | 0.0 | 0.0 | 0.0 | 0.33 | 0.0 | 0.0 | 0.0 | 0.0 | 0.0 | 0.0 | | | |
| A\|4241\|G | 0.0 | 0.0 | 0.0 | 0.12 | 0.0 | 0.0 | 0.0 | 0.0 | 0.0 | 0.0 | | | |
| C\|5051\|T | 0.0 | 0.0 | 0.0 | 0.12 | 0.0 | 0.0 | 0.0 | 0.0 | 0.0 | 0.0 | | | |
| A\|5177\|C | 0.0 | 0.0 | 0.0 | 0.14 | 0.0 | 0.0 | 0.0 | 0.0 | 0.0 | 0.0 | | | |
| C\|5178\|A | 0.0 | 0.0 | 0.0 | 0.26 | 0.0 | 0.0 | 0.0 | 0.0 | 0.0 | 0.0 | | | |
| C\|6145\|T | 0.0 | 0.0 | 0.0 | 0.12 | 0.0 | 0.0 | 0.0 | 0.0 | 0.0 | 0.0 | | | |
| T\|11343\|C | 0.0 | 0.0 | 0.0 | 0.12 | 0.0 | 0.0 | 0.0 | 0.0 | 0.0 | 0.0 | | | |
| A\|11430\|G | 0.0 | 0.0 | 0.0 | 0.2 | 0.0 | 0.0 | 0.0 | 0.0 | 0.0 | 0.0 | | | |
| C\|19983\|T | 0.0 | 0.0 | 0.0 | 0.0 | 0.0 | 0.0 | 0.0 | 0.0 | 0.24 | 0.0 | | | |
| T\|22156\|A | 0.0 | 0.0 | 0.0 | 0.11 | 0.0 | 0.0 | 0.0 | 0.0 | 0.0 | 0.0 | | | |
| C\|24797\|T | 0.0 | 0.0 | 0.0 | 0.11 | 0.0 | 0.0 | 0.0 | 0.0 | 0.0 | 0.0 | | | |
| C\|25587\|T | 0.0 | 0.0 | 0.0 | 0.0 | 0.0 | 0.0 | 0.0 | 0.0 | 0.15 | 0.0 | | | |
| T\|26324\|C | 0.0 | 0.0 | 0.0 | 0.11 | 0.0 | 0.0 | 0.0 | 0.0 | 0.0 | 0.0 | | | |
| C\|26333\|T | 0.0 | 0.0 | 0.0 | 0.83 | 0.0 | 0.0 | 0.0 | 0.0 | 0.0 | 0.0 | missense_variant | E | Thr30Ile |
| C\|26895\|T | 0.0 | 0.0 | 0.0 | 0.6 | 0.0 | 0.0 | 0.0 | 0.0 | 0.0 | 0.0 | | | |
| T\|2935\|C | 0.0 | 0.0 | 0.0 | 0.0 | 0.11 | 0.0 | 0.0 | 0.0 | 0.0 | 0.0 | | | |
| A\|7626\|G | 0.0 | 0.0 | 0.0 | 0.0 | 0.11 | 0.0 | 0.0 | 0.0 | 0.0 | 0.0 | | | |
| C\|14786\|T | 0.0 | 0.0 | 0.0 | 0.0 | 0.11 | 0.0 | 0.0 | 0.0 | 0.0 | 0.0 | | | |
| C\|21855\|T | 0.0 | 0.0 | 0.0 | 0.0 | 0.13 | 0.0 | 0.0 | 0.0 | 0.0 | 0.0 | | | |
| C\|18171\|T | 0.0 | 0.0 | 0.0 | 0.0 | 0.0 | 0.11 | 0.0 | 0.0 | 0.0 | 0.0 | | | |
| A\|22320\|C | 0.0 | 0.0 | 0.0 | 0.0 | 0.0 | 0.38 | 0.0 | 0.0 | 0.0 | 0.0 | | | |
| C\|27603\|T | 0.0 | 0.0 | 0.0 | 0.0 | 0.0 | 0.16 | 0.0 | 0.0 | 0.0 | 0.0 | | | |
| C\|28093\|T | 0.0 | 0.0 | 0.0 | 0.0 | 0.0 | 0.0 | 0.0 | 0.15 | 0.0 | No reads | | | |
| A\|28254\|C | 0.0 | 0.0 | 0.0 | 0.0 | 0.0 | 0.0 | 0.0 | 0.0 | 0.0 | 1.0 | missense_variant | ORF8 | Ile121Leu |
| C\|29750\|G | 0.0 | 0.0 | 0.0 | 0.0 | 0.0 | 0.11 | 0.0 | 0.0 | 0.0 | 0.0 | | | |
| C\|29754\|A | 0.0 | 0.0 | 0.0 | 0.0 | 0.0 | 0.12 | 0.0 | 0.0 | 0.0 | 0.0 | | | |
| T\|11647\|C | 0.0 | 0.0 | 0.0 | 0.0 | 0.0 | 0.0 | 0.0 | 0.25 | 0.0 | 0.0 | | | |
| G\|25429\|C | 0.0 | 0.0 | 0.0 | 0.0 | 0.0 | 0.0 | 0.0 | 0.36 | 0.0 | 0.0 | | | |
| T\|25586\|C | 0.0 | 0.0 | 0.0 | 0.0 | 0.0 | 0.0 | 0.0 | 0.13 | 0.0 | 0.0 | | | |
| C\|26388\|T | 0.0 | 0.0 | 0.0 | 0.0 | 0.0 | 0.0 | 0.0 | 0.25 | 0.0 | 0.0 | | | |
| C\|1913\|A | 0.0 | 0.0 | 0.0 | 0.0 | 0.0 | 0.0 | 0.0 | 0.11 | 0.0 | 0.0 | | | |
| T\|2112\|G | 0.0 | 0.0 | 0.0 | 0.0 | 0.0 | 0.0 | 0.0 | 0.16 | 0.0 | 0.0 | | | |
| G\|524\|A | 0.0 | 0.0 | 0.0 | 0.0 | 0.0 | 0.0 | 0.0 | 0.0 | 0.2 | 0.0 | | | |
| C\|17678\|T | 0.0 | 0.0 | 0.0 | 0.0 | 0.0 | 0.0 | 0.0 | 0.0 | 0.12 | 0.0 | | | |
| T\|18024\|C | 0.0 | 0.0 | 0.0 | 0.0 | 0.0 | 0.0 | 0.0 | 0.0 | 0.11 | 0.0 | | | |
| G\|25314\|T | 0.0 | 0.0 | 0.0 | 0.0 | 0.0 | 0.0 | 0.0 | 0.0 | 0.32 | 0.0 | | | |
| T\|10372\|C | 0.0 | 0.0 | 0.0 | 0.0 | 0.0 | 0.0 | 0.0 | 0.0 | 0.0 | 0.19 | | | |
| A\|12498\|G | 0.0 | 0.0 | 0.0 | 0.0 | 0.0 | 0.0 | 0.0 | 0.0 | 0.0 | 0.47 | | | |
| G\|12907\|T | 0.0 | 0.0 | 0.0 | 0.0 | 0.0 | 0.0 | 0.0 | 0.0 | 0.0 | 0.11 | | | |
| T\|16697\|C | 0.0 | 0.0 | 0.0 | 0.0 | 0.0 | 0.0 | 0.0 | 0.0 | 0.0 | 0.17 | | | |
| G\|19648\|A | 0.0 | 0.0 | 0.0 | 0.0 | 0.0 | 0.0 | 0.0 | 0.0 | 0.0 | 0.13 | | | |
| T\|20174\|G | 0.0 | 0.0 | 0.0 | 0.0 | 0.0 | 0.0 | 0.0 | 0.0 | 0.0 | 0.2 | | | |
| C\|5183\|T | 0.78 | 0.27 | 0.0 | 0.0 | 0.0 | 0.0 | 0.0 | 0.0 | 0.0 | 0.0 | | | |
| C\|635\|T | 0.0 | 0.0 | 0.0 | 0.0 | 0.0 | 0.35 | 0.33 | 0.0 | 0.0 | 0.0 | | | |
| G\|18670\|A | 0.0 | 0.0 | 0.51 | 0.0 | 0.12 | 0.0 | 0.0 | 0.0 | 0.0 | 0.0 | | | |
| C\|26029\|A | 0.0 | 0.0 | 0.0 | 0.0 | 0.0 | 0.13 | 0.15 | 0.0 | 0.0 | 0.0 | | | |
| G\|521\|T | 0.0 | 0.0 | 0.0 | 0.0 | 0.0 | 0.23 | 0.0 | 0.0 | 0.2 | 0.0 | | | |
| A\|23063\|T | 0.0 | 0.0 | 0.0 | 0.0 | 0.0 | 0.15 | 0.15 | 0.0 | 0.0 | 0.0 | | | |
| C\|26894\|T | 0.0 | 0.0 | 0.0 | 0.0 | 0.17 | 0.0 | 0.0 | 0.18 | 0.0 | 0.0 | | | |
| A\|3858\|G | 0.0 | 0.0 | 0.0 | 0.0 | 0.0 | 0.24 | 0.0 | 0.0 | 0.13 | 0.0 | | | |
| G\|4080\|A | 0.0 | 0.0 | 0.0 | 0.0 | 0.0 | 0.22 | 0.0 | 0.0 | 0.13 | 0.0 | | | |
| C\|19374\|T | 0.0 | 0.0 | 0.0 | 0.0 | 0.0 | 0.38 | 0.26 | 0.0 | 0.0 | 0.0 | | | |
| C\|25708\|T | 0.0 | 0.0 | 0.0 | 0.0 | 0.0 | 0.13 | 0.14 | 0.0 | 0.0 | 0.0 | | | |
| C\|28651\|T | 0.0 | 0.0 | 0.0 | 0.0 | 0.0 | 0.19 | 0.13 | 0.0 | 0.0 | 0.0 | | | |
| G\|26529\|C | 0.0 | 0.0 | 0.0 | 0.0 | 0.0 | 0.0 | 0.0 | 0.25 | 0.27 | 0.0 | | | |
| A\|28365\|T | 0.0 | 0.0 | 0.0 | 0.0 | 0.0 | 0.0 | 0.0 | 0.0 | 0.32 | 1.0 | missense_variant | N | Glu31Val |
| C\|14724\|T | 0.0 | 0.0 | 0.0 | 0.0 | 0.0 | 0.0 | 0.0 | 0.0 | 0.39 | 1.0 | synonymous_variant | ORF1ab | Phe4820Phe |
| C\|25844\|T | 0.0 | 0.23 | 0.0 | 0.0 | 0.0 | 0.14 | 0.16 | 0.0 | 0.0 | 0.0 | | | |
| A\|4085\|G | 0.0 | 0.0 | 0.0 | 0.0 | 0.0 | 0.0 | 0.18 | 0.87 | 0.36 | 0.0 | missense_variant | ORF1ab | Ile1274Val |
| C\|10369\|T | 0.0 | 0.0 | 0.0 | 0.0 | 0.0 | 0.0 | 0.15 | 0.85 | 0.37 | 0.0 | synonymous_variant | ORF1ab | Arg3368Arg |
| G\|15313\|A | 0.0 | 0.0 | 0.0 | 0.0 | 0.0 | 0.0 | 0.19 | 0.87 | 0.31 | 0.0 | missense_variant | ORF1ab | Ala5017Thr |
| C\|16726\|T | 0.0 | 0.0 | 0.0 | 0.0 | 0.0 | 0.0 | 0.21 | 0.88 | 0.4 | 0.0 | missense_variant | ORF1ab | His5488Tyr |
| C\|28948\|T | 0.0 | 0.0 | 0.0 | 0.0 | 0.0 | 0.0 | 0.17 | 0.69 | 0.28 | 0.0 | | | |
| A\|4094\|G | 0.0 | 0.0 | 0.0 | 0.0 | 0.0 | 0.0 | 0.3 | 0.0 | 0.49 | 0.93 | missense_variant | ORF1ab | Thr1277Ala |
| T\|12766\|A | 0.0 | 0.0 | 0.0 | 0.0 | 0.0 | 0.0 | 0.28 | 0.0 | 0.42 | 1.0 | missense_variant | ORF1ab | Asn4167Lys |
| C\|21057\|T | 0.0 | 0.0 | 0.0 | 0.0 | 0.0 | 0.0 | 0.31 | 0.0 | 0.5 | 1.0 | synonymous_variant | ORF1ab | Asp6931Asp |
| T\|21826\|A | 0.0 | 0.0 | 0.0 | 0.0 | 0.0 | 0.0 | 0.29 | 0.0 | 0.44 | 1.0 | missense_variant | S | Asp88Glu |
| C\|28969\|A | 0.0 | 0.0 | 0.0 | 0.0 | 0.0 | 0.0 | 0.5 | 0.0 | 0.54 | 0.92 | missense_variant | N | Ser232Arg |
| G\|29745\|T | 0.0 | 0.0 | 0.0 | 0.0 | 0.0 | 0.0 | 0.51 | 0.0 | 0.55 | 1.0 | downstream_gene_variant | S | |
| C\|28253\|T | 0.79 | 0.27 | 0.0 | 0.0 | 0.25 | 0.0 | 0.0 | 0.27 | 0.0 | 0.0 | | | |
| C\|18555\|T | 0.0 | 0.0 | 0.0 | 0.0 | 0.23 | 0.0 | 0.2 | 0.9 | 0.38 | 0.0 | synonymous_variant | ORF1ab | Asp6097Asp |
| G\|15530\|T | 0.0 | 0.0 | 0.0 | 0.0 | 0.0 | 0.0 | 0.29 | 0.11 | 0.49 | 1.0 | missense_variant | ORF1ab | Cys5089Phe |
| G\|3066\|T | 0.0 | 0.0 | 0.19 | 0.0 | 0.37 | 0.96 | 0.5 | 0.0 | 0.12 | 0.0 | missense_variant | ORF1ab | Gly934Val |
| G\|27235\|A | 0.0 | 0.0 | 0.21 | 0.0 | 0.35 | 0.82 | 0.24 | 0.0 | 0.13 | 0.0 | missense_variant | ORF6 | Ala12Thr |
| A\|23924\|G | 0.0 | 0.0 | 0.63 | 0.0 | 0.18 | 0.0 | 0.34 | 0.0 | 0.52 | 1.0 | missense_variant | S | Ile788Val |
| C\|24795\|T | 0.0 | 0.0 | 0.2 | 0.0 | 0.37 | 0.79 | 0.36 | 0.0 | 0.15 | 0.0 | | | |
| C\|5184\|T | 0.0 | 0.7 | 0.32 | 0.0 | 0.43 | 0.95 | 0.57 | 0.0 | 0.16 | 0.0 | missense_variant | ORF1ab | Pro1640Leu |
| C\|3330\|T | 0.0 | 0.0 | 0.68 | 0.0 | 0.58 | 0.0 | 0.45 | 0.98 | 0.82 | 1.0 | missense_variant | ORF1ab | Thr1022Ile |
| G\|5180\|A | 0.0 | 0.0 | 0.66 | 0.0 | 0.55 | 0.0 | 0.44 | 0.96 | 0.84 | 0.99 | missense_variant | ORF1ab | Asp1639Asn |
| C\|7165\|T | 0.0 | 0.0 | 0.66 | 0.0 | 0.55 | 0.0 | 0.49 | 0.98 | 0.88 | 0.86 | synonymous_variant | ORF1ab | Thr2300Thr |
| G\|11417\|T | 0.0 | 0.0 | 0.65 | 0.0 | 0.64 | 0.0 | 0.47 | 0.97 | 0.86 | 1.0 | missense_variant | ORF1ab | Val3718Phe |
| A\|12121\|T | 0.0 | 0.0 | 0.61 | 0.0 | 0.53 | 0.0 | 0.4 | 0.87 | 0.79 | 0.89 | synonymous_variant | ORF1ab | Pro3952Pro |
| C\|24138\|T | 0.15 | 0.23 | 0.11 | 0.0 | 0.23 | 0.0 | 0.12 | 0.62 | 0.35 | 0.0 | | | |
| C\|27925\|T | 0.14 | 0.21 | 0.0 | 0.0 | 0.24 | 0.15 | 0.44 | 0.76 | 0.93 | No reads | missense_variant | ORF8 | Thr11Ile |
| A\|4229\|G | 0.0 | 0.0 | 0.69 | 0.11 | 0.6 | 0.0 | 0.51 | 0.97 | 0.85 | 0.92 | missense_variant | ORF1ab | Thr1322Ala |
| C\|4230\|T | 0.0 | 0.29 | 0.29 | 0.19 | 0.4 | 0.96 | 0.49 | 0.0 | 0.15 | 0.0 | missense_variant | ORF1ab | Thr1322Ile |
| C\|241\|T | 1.0 | 1.0 | 1.0 | 1.0 | 1.0 | 1.0 | 1.0 | 1.0 | 1.0 | 1.0 | upstream_gene_variant | ORF1ab | |
| C\|3037\|T | 1.0 | 1.0 | 1.0 | 1.0 | 1.0 | 1.0 | 1.0 | 1.0 | 1.0 | 1.0 | synonymous_variant | ORF1ab | Phe924Phe |
| C\|14408\|T | 1.0 | 1.0 | 1.0 | 1.0 | 1.0 | 1.0 | 1.0 | 1.0 | 1.0 | 1.0 | missense_variant | ORF1ab | Pro4715Leu |
| A\|20268\|G | 1.0 | 1.0 | 1.0 | 1.0 | 1.0 | 1.0 | 1.0 | 1.0 | 1.0 | 1.0 | synonymous_variant | ORF1ab | Leu6668Leu |
| A\|23403\|G | 1.0 | 1.0 | 1.0 | 1.0 | 1.0 | 1.0 | 1.0 | 1.0 | 1.0 | 1.0 | missense_variant | S | Asp614Gly |

Common SNPs to all samples are shown in blue. Fixed SNPs are shown in dark orange (frequency> 0.8), intermediate variants (frequency 0.2-0.8) in light orange and minority variants (frequency <0.2) in light yellow.
