# Supplementary material for: Different Within-Host Viral Evolution Dynamics in Severely Immunosuppressed Cases with Persistent SARS-CoV-2
Source: Biomedicines. 2021 Jul 13;9(7):808. doi: 10.3390/biomedicines9070808 (PMC8301427; doi:10.3390/biomedicines9070808)
Supplement: Supplementary file 1 [file biomedicines-09-00808-s001.zip › biomedicines-1250640-SI/Supplementary Table_S2.pdf]

Patient A

	Nasopharyngeal	Nasopharyngeal	Nasopharyngeal	Nasopharyngeal	Nasopharyngeal	Nasopharyngeal	Nasopharyngeal	Nasopharyngeal	Nasopharyngeal			
	05/28/2020	06/17/2020	06/30/2020	07/15/2020	07/27/2020	08/06/2020	08/15/2020	09/06/2020	09/28/2020			
	66	86	99	114	126	136	145	167	189	Annotation	Gen	a.a change
C|18917|T	0.11	0.0	0.0	0.0	0.0	0.0	0.0	0.0	0.0			
G|25311|T	0.94	0.0	0.0	0.0	0.0	0.0	0.0	0.0	0.0	Non-synonymous_variant	S	Cys1250Phe
C|25413|T	0.14	0.0	0.0	0.0	0.0	0.0	0.0	0.0	0.0			
C|27942|T	0.5	0.0	0.0	no reads	no reads	no reads	no reads	no reads	no reads			
G|28221|T	0.0	0.0	0.78	0.0	0.0	0.0	0.0	0.0	0.0			
C|29546|T	0.0	0.0	0.0	0.0	0.0	0.0	0.0	0.0	0.0			
A|20323|G	0.0	0.0	0.0	0.0	0.0	0.0	0.0	0.0	0.0			
G|16141|T	0.0	0.13	0.0	0.0	0.0	0.0	0.0	0.0	0.0			
C|16575|T	0.0	0.0	0.2	0.0	0.0	0.0	0.0	0.0	0.0			
C|8208|A	0.0	0.0	0.0	0.11	0.0	0.0	0.0	0.0	0.0			
A|11342|T	0.0	0.0	0.0	0.13	0.0	0.0	0.0	0.0	0.0			
C|17939|T	0.0	0.0	0.0	0.13	0.0	0.0	0.0	0.0	0.0			
G|2293|T	0.0	0.0	0.0	0.0	0.0	0.18	0.0	0.0	0.0			
G|18498|A	0.0	0.0	0.0	0.0	0.0	0.19	0.0	0.0	0.0			
T|3952|C	0.0	0.0	0.0	0.0	0.0	0.0	0.0	0.0	0.15			
G|13520|A	0.0	0.0	0.0	0.0	0.0	0.0	0.0	0.12	0.0			
A|22759|G	0.0	0.0	0.0	0.0	0.0	0.0	0.0	0.13	0.0			
C|23525|T	0.0	0.0	0.0	0.0	0.0	0.0	0.0	0.0	0.22			
T|17732|G	0.0	0.0	0.0	0.0	0.0	0.0	0.0	0.0	0.19			
C|21846|T	0.0	0.26	0.0	0.0	0.0	0.0	0.0	0.0	0.0			
A|16438|G	0.0	0.13	0.0	0.0	0.0	0.0	0.0	0.0	0.0			
G|25691|A	0.0	0.23	0.0	0.0	0.0	0.0	0.0	0.0	0.0			
C|5585|A	0.0	0.0	0.0	0.0	0.0	0.0	0.0	0.15	0.15			
T|17688|C	0.0	0.0	0.0	0.0	0.0	0.0	0.0	0.12	0.12			
C|9693|T	0.0	0.0	0.0	0.0	0.0	0.0	0.0	0.18	0.15			
C|22591|T	0.0	0.0	0.0	0.0	0.0	0.0	0.0	0.16	0.16			
A|28254|C	0.0	0.0	0.21	0.0	0.0	0.64	0.48	0	0			
G|14118|A	0.0	0.0	0.0	0.0	0.0	0.0	0.53	0.52	0.61			
C|13326|T	0.0	0.0	0.0	0.0	0.0	0.0	0.55	0.44	0.6			
C|24378|T	0.0	0.0	0.0	0.0	0.0	0.0	0.53	0.47	0.62			
C|28977|T	0.0	0.0	0.0	0.0	0.0	0.24	0.53	0.43	0.39			
A|20481|G	0.0	0.0	0.0	0.0	0.0	0.16	0.19	0.4	0.31			
C|12068|T	0.0	0.0	0.0	0.0	0.95	0.81	0.69	0.52	0.61	synonymous_variant	ORF1ab	Leu3935Leu
C|14520|T	0.0	0.0	0.0	0.0	0.94	0.8	0.66	0.52	0.63	synonymous_variant	ORF1ab	Asn4752Asn
T|20427|G	0.0	0.0	0.0	0.0	0.92	0.8	0.76	0.57	0.61	Non-synonymous_variant	ORF1ab	Ile6721Met
T|22076|G	0.0	0.0	0.0	0.0	0.93	0.71	0.62	0.47	0.57	Non-synonymous_variant	S	Ser172Ala
G|24193|C	0.0	0.0	0.0	0.13	0.98	1.0	1.0	1.0	1.0	synonymous_variant	S	Leu877Leu
G|24751|A	0.0	0.0	0.0	0.92	1.0	1.0	1.0	1.0	1.0	synonymous_variant	S	Leu1063Leu
G|28198|C	0.0	0.0	0.0	0.11	0.97	?	1.0	0.97	1.0	Non-synonymous_variant	ORF8	Cys102Ser
C|4776|T	0.0	0.0	0.34	1.0	1.0	1.0	1.0	1.0	1.0	Non-synonymous_variant	ORF1ab	Thr1504Ile
T|15835|C	0.0	0.0	0.29	0.96	0.96	0.96	0.44	0.45	0.34	Non-synonymous_variant	ORF1ab	Cys5191Arg
C|19944|T	0.0	0.0	0.37	1.0	1.0	1.0	1.0	1.0	1.0	synonymous_variant	ORF1ab	Ala6560Ala
C|7239|T	0.0	0.67	0.91	1.0	1.0	1.0	1.0	1.0	1.0	Non-synonymous_variant	ORF1ab	Ala2325Val
T|27634|C	0.0	0.2	0.93	1.0	1.0	1.0	1.0	1.0	1.0	Non-synonymous_variant	ORF7a	Ser81Pro
T|5406|C	0.98	0.79	1.0	1.0	1.0	1.0	1.0	1.0	1.0	Non-synonymous_variant	ORF1ab	Ile1714Thr
C|9438|T	0.98	0.76	0.99	1.0	1.0	1.0	1.0	1.0	1.0	Non-synonymous_variant	ORF1ab	Thr3058Ile
C|12974|T	0.98	0.73	0.98	1.0	1.0	1.0	1.0	1.0	1.0	synonymous_variant	ORF1ab	Leu4237Leu
T|21752|A	0.98	0.74	1.0	1.0	1.0	1.0	1.0	1.0	1.0	Non-synonymous_variant	S	Trp64Arg
G|11083|T	1.0	1.0	1.0	1.0	1.0	1.0	1.0	1.0	1.0	Non-synonymous_variant	ORF1ab	Leu3606Phe
G|26144|T	1.0	1.0	1.0	1.0	1.0	1.0	1.0	1.0	1.0	Non-synonymous_variant	ORF3a	Gly251Val

Common SNPs to all samples are shown in blue. Fixed SNPs are shown in dark orange (frequency> 0.8), intermediate variants (frequency 0.2-0.8) in light orange and minority variants (frequency <0.2) in light yellow.
