# Supplementary material for: Different Within-Host Viral Evolution Dynamics in Severely Immunosuppressed Cases with Persistent SARS-CoV-2
Source: Biomedicines. 2021 Jul 13;9(7):808. doi: 10.3390/biomedicines9070808 (PMC8301427; doi:10.3390/biomedicines9070808)
Supplement: Supplementary file 1 [file biomedicines-09-00808-s001.zip › biomedicines-1250640-SI/Supplementary_Table_S4.pdf]

	Nasopharyngeal	Plasma	Nasopharyngeal	Nasopharyngeal	Nasopharyngeal	Nasopharyngeal	Bronchoaspirate			
	0	17	21	49	56	66	66			
	04/27/2020	05/14/2020	05/18/2020	06/15/2020	06/22/2020	07/02/2020	07/02/2020	Annotation	Gen	a.a change
C|11074|T	0.0	0.0	0.0	0.0	0.0	0.0	0.0			
C|29635|T	0.0	0.3	0.0	0.0	0.0	0.0	0.0			
T|7012|G	0.0	0.14	0.0	0.0	0.0	0.0	0.0			
T|20697|C	0.0	0.13	0.0	0.0	0.0	0.0	0.0			
T|25969|C	0.0	0.27	0.0	0.0	0.0	0.0	0.0			
C|26261|T	0.0	0.26	0.0	0.0	0.0	0.0	0.0			
A|29643|G	0.0	0.15	0.0	0.0	0.0	0.0	0.0			
C|11674|T	0.0	0.0	0.0	0.0	0.18	0.0	0.0			
C|16375|T	0.0	0.0	0.0	0.0	0.0	0.0	0.0			
C|5812|T	0.0	0.0	0.0	0.0	0.22	0.0	0.0			
G|9203|A	0.0	0.0	0.0	0.0	0.0	0.17	0.0			
T|9806|G	0.0	0.0	0.0	0.12	0.0	0.0	0.0			
C|12781|T	0.0	0.0	0.0	0.0	0.0	0.15	0.0			
G|14831|A	0.0	0.0	0.0	0.0	0.21	0.0	0.0			
G|15769|T	0.0	0.0	0.0	0.0	0.0	0.21	0.0			
C|19550|T	0.0	0.0	0.0	0.0	0.0	0.26	0.0			
C|21648|T	0.0	0.0	0.0	0.0	0.0	0.22	0.0			
C|23277|T	0.0	0.0	0.0	0.0	0.0	0.22	0.0			
G|23402|A	0.0	0.0	0.0	0.0	0.0	0.14	0.0			
A|25470|T	0.0	0.0	0.0	0.25	0.0	0.0	0.0			
C|27925|T	0.0	0.0	0.0	0.0	0.17	0.0	0.0			
C|6983|T	0.0	0.0	0.0	0.0	0.0	0.12	0.0			
T|28243|C	0.0	0.0	0.0	0.0	0.0	0.11	0.0			
C|28253|T	0.0	0.0	0.0	0.0	0.0	0.12	0.0			
C|28887|T	0.0	0.0	0.0	0.0	0.0	0.11	0.0			
A|696|G	0.0	0.0	0.0	0.0	0.0	0.0	0.94	Non_synonymous	ORF1ab	Asp144Gly
A|2240|G	0.0	0.0	0.0	0.0	0.0	0.0	0.92	Non_synonymous	ORF1ab	Ile659Val
C|7173|T	0.0	0.0	0.0	0.0	0.0	0.0	0.95	Non_synonymous	ORF1ab	Ser2303Phe
C|10790|T	0.0	0.0	0.0	0.0	0.0	0.13	0.0			
G|15734|A	0.0	0.0	0.0	0.0	0.0	0.3	0.0			
T|18471|C	0.0	0.0	0.0	0.0	0.0	0.11	0.0			
T|18849|C	0.0	0.0	0.0	0.0	0.0	0.15	0.0			
A|19652|G	0.0	0.0	0.0	0.0	0.0	0.31	0.0			
C|19875|T	0.0	0.0	0.0	0.0	0.0	0.18	0.0			
A|20129|G	0.0	0.0	0.0	0.0	0.0	0.32	0.0			
G|21600|T	0.0	0.0	0.0	0.0	0.0	0.18	0.0			
G|23587|C	0.0	0.0	0.0	0.0	0.0	0.16	0.0			
T|26879|G	0.0	0.0	0.0	0.0	0.0	0.19	0.0			
C|26895|T	0.0	0.0	0.0	0.0	0.0	0.0	0.73			
A|26927|G	0.0	0.0	0.0	0.0	0.0	0.29	0.0			
G|29266|C	0.0	0.0	0.0	0.0	0.0	0.16	0.0			
G|29825|C	0.0	0.0	0.0	0.0	0.0	0.19	0.0			
C|14016|A	0.0	0.0	0.0	0.0	0.0	0.0	0.18			
T|15715|G	0.0	0.0	0.0	0.0	0.0	0.0	0.64			
G|21468|T	0.0	0.0	0.0	0.0	0.0	0.0	0.19			
C|17536|T	0.0	0.0	0.44	0.44	0.0	0.0	0.0			
A|23403|G	0.0	0.0	0.0	0.35	0.23	0.0	0.0			
T|28382|C	0.0	0.0	0.0	0.0	0.14	0.23	0.0			
C|16466|T	0.0	0.0	0.35	0.0	0.0	0.31	0.0			
G|14414|T	0.0	0.0	0.0	0.44	0.64	0.3	0.0			
C|23525|T	0.0	0.0	0.0	0.43	0.64	0.64	0.0			
C|28830|T	0.0	0.0	0.0	0.47	0.62	0.29	0.0			
G|15814|A	0.0	0.0	0.0	0.39	0.64	0.34	0.96	Non_synonymous	ORF1ab	Val5184Ile
C|4551|T	1.0	1.0	1.0	1.0	1.0	1.0	1.0	synonymous_variant	S	Glu773Glu
C|8782|T	1.0	1.0	1.0	1.0	1.0	1.0	1.0	Non_synonymous	S	Gln1071Leu
C|13115|T	1.0	1.0	1.0	1.0	1.0	1.0	1.0	Non_synonymous	ORF3a	Val50Ala
A|23881|G	1.0	1.0	1.0	1.0	1.0	1.0	1.0	synonymous_variant	ORF3a	Ile232Ile
A|24774|T	0.94	0.94	0.94	0.93	0.87	0.87	0.93	Non_synonymous	ORF1ab	Thr1429Ile
T|25541|C	1.0	1.0	1.0	1.0	1.0	1.0	1.0	Non_synonymous	ORF8	Thr87Ile
C|26088|T	1.0	1.0	1.0	1.0	1.0	1.0	1.0	synonymous_variant	ORF1ab	Ser2839Ser
T|28144|C	1.0	1.0	1.0	1.0	1.0	1.0	0.99	synonymous_variant	ORF1ab	Leu4284Leu
C|28153|T	0.95	0.95	0.97	0.88	0.9	0.93	0.87	Non_synonymous	ORF8	Leu84Ser
G|29254|T	1.0	1.0	1.0	1.0	1.0	1.0	1.0	synonymous_variant	N	Ser327Ser
