# Supplementary material for: Different Within-Host Viral Evolution Dynamics in Severely Immunosuppressed Cases with Persistent SARS-CoV-2
Source: Biomedicines. 2021 Jul 13;9(7):808. doi: 10.3390/biomedicines9070808 (PMC8301427; doi:10.3390/biomedicines9070808)
Supplement: Supplementary file 1 [file biomedicines-09-00808-s001.zip › biomedicines-1250640-SI/SupplMethods.pdf]

Whole genome sequencing

Eleven microliters of RNA were used as template for reverse transcription with Invitrogen SuperScript IV reverse transcriptase (ThermoFisher Scientific, Massachusetts, USA) and random hexamers (ThermoFisher Scientific, Massachusetts, USA). Whole genome amplification of the coronavirus was done using the Artic_nCov-2019_V3 panel of primers (Integrated DNA Technologies, Inc., Coralville, Iowa, USA) (artic.network/ncov-2019) and the Q5 Hot Start DNA polymerase enzyme (New England Biolabs, Ipswich, Massachusetts, USA). Libraries were prepared using the Nextera Flex DNA Library Preparation Kit (Illumina lnc, California, USA) following the manufacturer´s instructions and quantified with the Quantus™ Fluorometer (Promega, Wisconsin, USA), before being pooled at an equimolar concentration (4 nM). Next, libraries were sequenced in pools of up to 17 libraries on the Miseq system (Illumina Inc, California, USA) using the MiSeq Reagent Micro kit v2 (2x151pb) or in pools of up to 96 libraries with the MiSeq reagent (2x201 pb).

Fasta files above the GISAID thresholds were deposited in GISAID :

EPI_ISL_778644, EPI_ISL_510115, EPI_ISL_654337, EPI_ISL_654340, EPI_ISL_654338, EPI_ISL_654336, EPI_ISL_510148, EPI_ISL_654186, EPI_ISL_654166, EPI_ISL_654191, EPI_ISL_654182, EPI_ISL_654172, EPI_ISL_654173, EPI_ISL_654170, EPI_ISL_654194, EPI_ISL_778645, EPI_ISL_777902, EPI_ISL_780026, EPI_ISL_776877, EPI_ISL_776875, EPI_ISL_780023, EPI_ISL_776871, EPI_ISL_776870, EPI_ISL_776772, EPI_ISL_776770.

An in-house analysis pipeline was used to analyse the sequencing reads (access at https://github.com/pedroscampoy/covid_multianalysis). Pipeline steps in brief: 1)

removal of human reads with Kraken [https://genomebiology.biomedcentral.com/articles/10.1186/gb-2014-15-3-r46]; 2) pre-processing and quality assessment of the fastq files with fastp v0.20.1 [https://academic.oup.com/bioinformatics/article/34/17/i884/5093234] (arguments: --cut tail, --cut-window-size, --cut-mean-quality , -max_len1 ,-max_len2) and fastQC v0.11.9 [Andrews S.; S Bittencourt a, "FastQC: a quality control tool for high throughput sequence data – ScienceOpen," *Babraham Inst.*, p. http://www.bioinformatics.babraham.ac.uk/projects/, 2010.]; 3) mapping with bwa v0.7.17 [H. Li and R. Durbin, "Fast and accurate short read alignment with Burrows-Wheeler transform," *Bioinformatics*, vol. 25, no. 14, pp. 1754–1760, 2009.] and variant calling using IVAR v1.2.3 [https://genomebiology.biomedcentral.com/articles/10.1186/s13059-018-1618-7] using Wuhan-1 sequence (NC_045512.2) as reference; 4) Recalibration of low coverage positions using joint variant calling.

To prevent spurious variant calling, only alternative alleles with a frequency > 10% were considered in the ensuing analyses.

The following frequencies were considered for variant calling: minority variants (MVs; frequency < 20%), intermediate variants (IVs; frequency 20%-80%), and fixed single nucleotide polymorphisms (SNPs; frequency > 80%).

Short tandem repeat analysis

Human identity testing analysis was performed by short tandem repeat (STR) PCR (Mentype® Chimera® Biotype, Germany) on the same specimens used to perform SARS-CoV-2 RT-PCRs and sequencing. We examined 12 non-coding STR loci and the gender-specific locus amelogenin (Supplementary Table), labelled with three different dyes (6-

FAM™, BTG, and BTY). The selected loci offer a very high rate of heterozygosity and balanced allelic distribution (Thiede et al., 2004). PCRs were performed with 0.2-1 ng of genomic DNA using the Mentype® Chimera® PCR amplification kit (Biotype, Germany), the GeneAmp® PCR System 9700 Thermal Cycler (Applied Biosystems), and subsequent capillary electrophoresis in the Genetic Analyzer 3130*xl* (Applied Biosystems) under the conditions recommended by the manufacturer.

Cell Cultures

For viral amplification, Vero E6 cells (ATCC CRL1586) were seeded on a 96-well plate (Greiner #3599) at 2e5 cells/mL in Dulbecco´s modified eagle medium (Gibco) supplemented with 10% foetal bovine serum (Gibco) and 2% penicillin/streptomycin/amphotericin B Solution (Sigma) the day before the infection. Vero E6 cells were inoculated with 50 µL of samples and the plates incubated at 37 ºC and 5% CO2 for five days; the cytopathic effect (CPE) was analysed every two hours with an IncuCyte automated microscope (Sartorious), using phase image at 10X objective, four fields per well. For nucleic acid extraction we used the KingFisher™ Flex (Thermo Scientific) Purification System and the MagMax Viral/pathogen Nucleic Acid Isolation Kit (Applied Biosystem) and TaqPath COVID-19 CE-IVD RT-PCR Kit (Applied Biosystems) for RT-PCR to quantify SARS-CoV-2, following the manufacturers guidelines. Serial dilutions of SARS CoV 2 (coronavirus strain 2019-nCoV/USA-WA/2020, BEI) were used as controls for the CPE and RT-PCR analyses.

Experimental procedures with SARS-CoV-2 were carried out following standard operating procedures in compliance with biosafety level 3 (BSL3) regulations. Biological samples were treated according to GSK policies for the manipulation of human biological samples.

Cultures were considered positive when fulfilling at least one of the following criteria i) appearance of CPE, ii) positive immunofluorescence detected, or iii) a decrease of ≥ 3 (equivalent to a 1 log increase in virus quantity) between the Ct of the original sample and final culture supernatant.

Subgenomic RNA

RNA remnants purified (KingFisher, Thermo Fisher Scientific, Waltham, Massachusetts) from diagnostic nasopharyngeal swab specimens (300 µl of UTM swabs, COPAN, Biomerieux; Marcy-l'Étoile, France) were used as templates for the PCR design described elsewhere (11) for specific detection of the SG E gene RNA (+ strand). Viral genomic RNA for the E gene (+ strand) and/or a human housekeeping gene (RNAse P) were detected as controls.
